# Mission possible: an optimised protocol for the unbarcodable Ceraphronoidea (Hymenoptera)

**DOI:** 10.3897/BDJ.10.e84860

**Published:** 2022-06-30

**Authors:** Cristina Vasilita, Marina Moser, Lars Krogmann

**Affiliations:** 1 State Museum of Natural History, Stuttgart, Germany State Museum of Natural History Stuttgart Germany; 2 University of Hohenheim, Stuttgart, Germany University of Hohenheim Stuttgart Germany

**Keywords:** GBOLIII, dark taxa, COI, Ceraphronoidea, barcoding, PCR

## Abstract

DNA barcodes provide a reliable and efficient solution to resolving cryptic species complexes and accelerate species discoveries. The superfamily Ceraphronoidea (Hymenoptera) is a group of parasitoid wasps for which a barcoding approach could be of great help, if it were not for the very poor results. The inability to obtain barcodes for the majority of treated ceraphronoids halts progress on the taxonomy of this hyperdiverse parasitoid group. We here present a working protocol for the barcoding of ceraphronoid wasps which yields a first-time over 90% success rate.

## Introduction

Despite being one of the most abundant groups of microhymenoptera recovered by various collecting efforts, be it Malaise trap or sweep netting (Martinez de Murguia et al. 2001), Ceraphronoidea is still severely understudied. Only a fraction of their species diversity has been described and any large-scale biodiversity assessment that involved ceraphronoid wasps (Ceraphronidae and Megaspilidae) were not very successful (GBOL I & II, NorBOL, SweBOL). One of the reasons behind this is the poor results obtained by molecular approaches using standard protocols. Ceraphronoidea stands out amongst all hyperdiverse lineages of Microhymenoptera due to their low DNA barcoding success rate. This is reflected in the extremely low number of barcodes available in BOLD (26 species out of the ~ 660 described and over 1200 unassociated BINs). Additionally, in the two previous phases of the German Barcode of Life (GBOL) Project, only 28.5% of all extractions carried out for Ceraphronoidea resulted in DNA barcodes.

The unavailability of DNA barcodes hinders progress on the taxonomy of Ceraphronoidea, as barcoding would be essential for species delimitation in this group. External morphology alone is not sufficient to diagnose species as characters tend to be monotonous throughout the superfamily and often affected by allometry. The two taxonomically useful morphological characteristics of this group are the male genitalia (Mikó et al. 2013) and the Waterston’s evaporatorium (Ulmer et al. 2021). While effective in species delimitation, these two character complexes have their limits, the first one being confined to one sex and the latter referring to just one of the two families - Ceraphronidae. Matching the two sexes is an additional challenge due to the strong sexual dimorphism.

This fact, paired with the tiny size and monotonous, often uninformative external morphology of ceraphronoid wasps, results in what we call a "dark taxon" (Page 2016). The third phase of the German Barcode of Life project (GBOLIII: dark taxa) focuses specifically on these kinds of taxonomic impediments in two very diverse and understudied insect orders: Diptera and Hymenoptera (Hausmann et al. 2020). Ceraphronoidea was included in GBOLIII with the aim to unravel its true diversity and to understand ecosystem linkages as a prerequisite for practical and sustainable conservation programmes.

**Figure 1.**
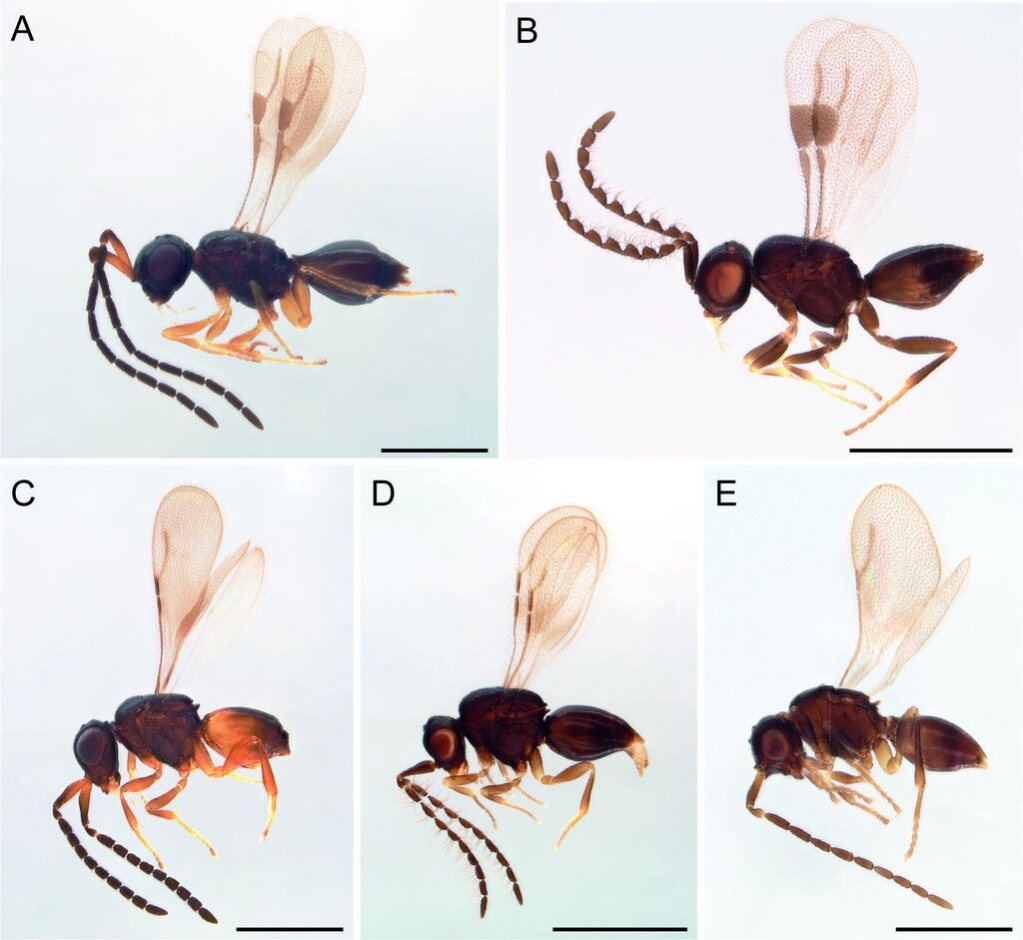
Voucher specimens of Ceraphronoidea after DNA extraction. Scale bar: 500 µm. (A) *Conostigmus* sp. (Megaspilidae), male. (B) *Dendrocerus* sp. (Megaspilidae), male. (C) *Ceraphron* sp. (Ceraphronidae), male. (D) *Aphanogmus* sp. (Ceraphronidae), male. (E) *Lagynodespallidus* (Boheman, 1832) (Megaspilidae), male.

The initial goal was to develop a functional barcoding protocol for Ceraphronoidea. The first challenge is posed by the small size of the animals, especially in ceraphronids, which translates into reduced amounts of extracted DNA material. The biology of these wasps is another factor that might negatively affect the barcoding results. By being parasitoid, a ceraphronoid wasp will feed on its host until it reaches adulthood, which means that its gut is filled with host tissue in various stages of molecular digestion. When that is the case, the use of non-specific primers might result either in the amplification of DNA originating from both the host and the parasitoid or, occasionally, from the host alone. This kind of data is extremely useful and can be exploited in exploring host-parasitoid linkages (Rougerie et al. 2011), but it can also cause problems when the parasitoid barcode is the target. Another aspect of ceraphronoid biology that should be considered is their cohabitation with endosymbiont bacteria, such as *Wolbachia* (West et al. 1998). More often than desired, COI sequences turn out to be of *Wolbachia* or *Rickettsia* and, when that happens, there is little to be done to salvage the sample. Lastly, a non-destructive method of DNA extraction should be used so that the sclerotised body of the insect could be retrieved and used for subsequent morphological analyses; therefore, the vouchers should be as intact as possible and permanently stored in an accessible natural history collection (Fig. 1).

In this publication, we provide a step-by-step solution and present an optimised barcoding protocol for the previously "unbarcodable" Ceraphronoidea which yields a first-time high success rate (> 80%).

## Material and methods

Non-destructive DNA extraction was performed either by the use of the Qiagen Blood & Tissue Kit following manufacturer’s protocol with minor alterations as in Cruaud et al. (2019) or following the GBOL protocol developed in-house that employs a Xiril Automatic Workstation; details regarding the extraction method performed on each sample can be consulted in Suppl. material 1.

The first testing batch consisted of 12 samples, specimens from Ceraphronidae and Megaspilidae both. DNA quantity in the eluate was checked prior to PCR reactions using an Implen NanoPhotometer N60. Amplification of the mitochondrial COI was attempted by the use of several primer pairs (Table 1) in 25 µl PCR reaction with 4 µl DNA template, cycler conditions set accordingly (Table 2).

FastGene Optima HotStart Ready Mix was used for all PCR reactions. PCR optimisation was conducted by temperature gradient PCR, by increasing the quantity of the DNA template or by adding trehalose to the reaction (Spiess et al. 2004). Sometimes, two or all three of these approaches were carried out simultaneously. The success of the PCR reaction was assessed by agarose gel electrophoresis. Double read Sanger sequencing was performed on samples with a positive PCR result and sequences were assembled, trimmed and blasted using Geneious Prime 2022.0.1.

## Results and Discussion

In the test batch of twelve specimens, the DNA concentration was between 2.65 and 10.9 ng/µl (for DNA concentrations of all processed samples, see Suppl. material 1). The use of Folmer primers was attempted, but failed, as no amplification could be detected in any of the samples and our attempts at optimising the PCR protocol did not change the outcome. Folmer primers can still be used when treating Ceraphronoidea, but from our experience, they are not the best choice. The use of the Lepidoptera primers yielded better PCR results (50%, n = 12); unfortunately, four out of the six samples sequenced turned out to be of *Wolbachia* sp., one of them beings a cecidomyiid (Diptera) DNA and just one sequence matched with sequences of unidentified Ceraphronidae. The trace files of the non-ceraphronoid sequences showed no indication of the presence of another organism’s genome in the eluate (i.e. the traces were clean and peaks were unique). This leads to the conclusion that the primers were very unlikely to attach to the DNA strands of the wasp, if there were any in the extract.

We decided to target a shorter region of the barcode, in case the main reason of consequent failures was the state of fragmentation of the DNA strands. For this purpose, the COI_pF2 primer was paired with the reverse HCO2198 primer which should produce a sequence with a length of ~ 450 nucleotides. This protocol is usually used as a last resort in The Research Group of Invertebrate Diversity and Phylogeny in Iași, Romania (first author’s former lab) in cases where DNA fragmentation is presumed to be the reasoning behind poor results and has been successfully tested in Scelionidae, Eupelmidae and Ichneumonidae (Lucian Fusu & Madalina Viciriuc, pers. comm.). This attempt resulted in very good PCR (91.6%, n = 12) and sequencing results (91.6%, n = 12). According to BLASTn, all of the obtained sequences matched unidentified species of Ceraphronidae or Megaspilidae. By ruling out the option of the absence of any parasitoid DNA in the extracts, the only reasonable explanation for the failed attempts at barcoding was that the DNA was either in an advanced state of fragmentation or there was an issue at the binding site of the forward LCO1490 and LepF primers. The first hypothesis was tested by pairing the COI_pF2 primer with the COI_2437d reverse one which should result in the amplification of the slightly longer, ~ 870 bp fragment. We obtained a 91.6% sequencing success rate in the test batch and the same approach was subsequently used on a higher number of samples (n = 46). The PCR reaction resulted in the amplification of 41 samples out of 46 attempted, producing 33 clean sequences belonging to Ceraphronidae (the sequencing failed in six reactions and showed contamination in two others). Nonetheless, a 71.7% success rate was significantly better than anything obtained in the previous stages of the GBOL project. The only downside of this workflow was that it produced sequences overlapping with the standard barcode region only on ~ 450 nucleotides, as the COI_pF2 primer was placed ~ 200 nucleotides downstream from the LCO1490/LepF binding site.

**Figure 2.**
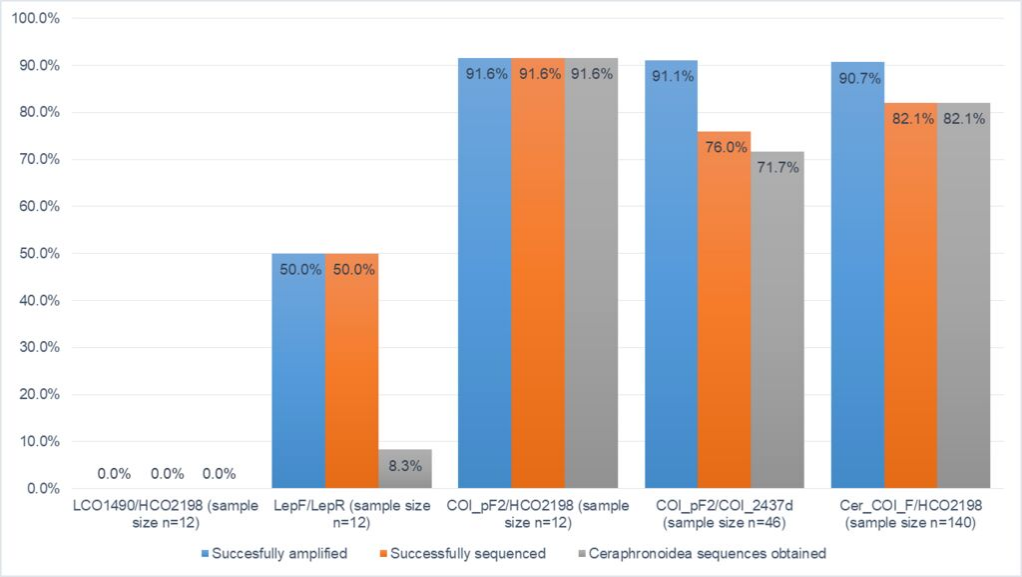
Graphical display of the PCR and sequencing success rate by using different sets of primers.

The positive outcome of the COI_pF2/COI_2437d attempt left only one possible cause for our low rates of amplification: there was indeed a mismatch between the two most commonly used forward primers (LCO1490 and LepF) and the binding site in at least some Ceraphronoidea. Using sequences available from Genbank, we designed a new forward primer Cer_COI_F: GSTTTATGAGCHGGAATANTAGG positioned downstream from the classic forward primers. The optimal annealing temperature was determined to be 53°C by temperature gradient PCR. By pairing the newly-designed primer with the reverse HCO2198, we achieved a 100% PCR and sequencing success in our test batch. The new forward primer was ultimately tested on a larger batch of samples (n = 140) and the barcoding success rate dropped to 82.1%. It is also notable to mention that the quality estimate exceeded 95% in all obtained sequences but nine (all sequences were of quality over 90%). Unfortunately, the use of the newly-designed forward primer, paired with the HCO2198, shortens the barcodes to a final length of 617 nucleotides, but considering the significant rise in efficiency, from ca. 30% to over 80% success rate (Fig. 2), we believe it to be a fair trade.

## Conclusions

In the world of standard operating procedure and pipeline workflows, it is important to remember that a successful protocol for one group or another does not equate to a universal procedure and might not yield similar or any results at all when applied somewhere else. We were reminded through the course of our work here that a protocol might need to be tailored for the specifics of a group. Additionally, even though a pipeline approach is more appealing, at least from the time investment point of view, it is clear that sometimes the benefit might be overruled by the poor success rate. The protocol we provide is functional for the molecular treatment of ceraphronoids. We hope that by providing a solution to an anecdotally "unbarcodable" taxon, we will accelerate species discovery and aid further exploration of ceraphronoid wasps.

## Supplementary Material

C74BAFAF-D1F7-5E02-8733-5D294C9B8B97 10.3897/BDJ.10.e84860.suppl1 Supplementary material 1 Supplementary data Data type PCR results and barcode quality Brief description Details regarding the extraction method and PCR primers used on every specimen, including the quality of the obtained sequences, with notes on failed reactions. File: oo_663357.xlsx https://binary.pensoft.net/file/663357 Cristina Vasilita

## Figures and Tables

**Table 1.** Primers used for amplification.

Primer name	Direction	Primer sequence 5-3	Reference
LCO1490	F	GGTCAACAAATCATAAAGATATTGG	Folmer et al. (1994)
HCO2198	R	TAAACTTCAGGGTGACCAAAAAATCA	Folmer et al. (1994)
COI_pF2	F	ACCWGTAATRATAGGDGGDTTTGGDAA	Simon et al. (1994)
COI_2437d	R	GCTARTCATCTAAAWAYTTTAATWCCWG	Kaartinen et al. (2010)
LepF	F	ATTCAACCAATCATAAAGATATTGG	Hajibabaei et al. (2006)
LepR	R	TAAACTTCTGGATGTCCAAAAAATCA	Hajibabaei et al. (2006)
Cer_COI_F	F	GSTTTATGAGCHGGAATANTAGG	herein

**Table 2.** PCR conditions.

Primer pair	Thermocycler conditions
LCO1490/HCO2198 LepF/LepR COI_pF2/COI_2437d	94° for 2’, (96° for 1’, 45° for 1’, 72° for 1’30’’) - 5 cycles, (93° for 1’, 50° for 1’, 72° for 1’30’’) - 35 cycles, 72° for 5’
COI_pF2/HCO2198	94° for 2’, (96° for 1’, 45° for 45’’, 72° for 1’30’’) - 5 cycles, (93° for 1’, 50° for 45’’, 72° for 1’30’’) - 35 cycles, 72° for 5’
Cer_COI_F/HCO2198	94° for 2’, (96° for 1’, 48° for 1’, 72° for 1’30’’) - 5 cycles, (93° for 1’, 53° for 1’, 72° for 1’30’’) - 35 cycles, 72° for 5’
